# Aminoglycoside use in renal failure

**DOI:** 10.4103/0971-4065.70839

**Published:** 2010-07

**Authors:** S. Nayak-Rao

**Affiliations:** Department of Nephrology, Bahrain Specialist Hospital, PO Box 10588, Manama, Bahrain

**Keywords:** Aminoglycosides, clearance, hemodialysis, therapeutic drug levels

## Abstract

Aminoglycosides are the mainstay in the treatment of serious gram negative infections including catheter-associated infections. They are not metabolized and are rapidly excreted as such by glomerular filtration resulting in a plasma t^½^ of approximately two hours in those with normal renal function. The t^½^, however, can extend to 30-60 hours in patients who are functionally anephric; therefore, dosage reduction or modification is necessary in renal failure patients. In patients on hemodialysis the clearance of aminoglycosides is significant and variable. The concept of conventional postdialysis dosing in patients on hemodialysis needs to be revised in favor of higher predialysis doses to maintain effective bactericidal activity. This article is a brief review of the use of aminoglycosides in renal failure patients.

## Introduction

Aminoglycosides have long been important in the treatment of serious gram negative infections. They have bactericidal activity against most gram negative bacteria including *Acinetobacter*, *Enterobacter*, *Escherichia coli*, *Klebsiella*, *Proteus*, *Salmonella*, *Pseudomonas*, *Serratia*, and *Shigella*. They also act synergistically against gram positive organisms such as *Staphylococcus aureus* and *Staphylococcus* epidermidis. Energy is needed for aminoglycoside uptake into the bacterial cell and intracellular transport is oxygen dependant; hence, anaerobes are resistant to aminoglycosides.

## Pharmacokinetics

The antibacterial properties of aminoglycosides are believed to result from inhibition of bacterial protein synthesis through irreversible binding to the 30s bacterial ribosome.[1] Later experimental studies, however, showed that the initial site of action is the outer bacterial cell membrane. The cationic antibiotic molecules create fissures in the bacterial cell wall resulting in leakage of intracellular contents and enhanced antibiotic uptake.

When given in intravenous infusion over 30-60 mins, amino glycosides follow a three compartment pharmacokinetic model of: Distribution (α), elimination (β), and tissue release (γ). The gamma phase begins approximately 16 hrs postinfusion when the tissue bound drug is released. The pharmacodynamic properties of aminoglycosides are the following:[2,3]

- Concentration dependant killing
- Significant postantibiotic effect

Aminoglycosides are not metabolized and are rapidly excreted by glomerular filtration resulting in a plasma t^½^ of approximately 2 hrs in those with normal renal function. The t^½^, however, can extend to 30-60 hrs in patients who are functionally anephric.[2] The half-life of aminoglycosides in the renal cortex is approximately 100 hrs, and therefore repetitive dosing results in renal cortical accumulation and ensuing toxicity. The risk of nephrotoxicity is enhanced by certain risk factors mentioned below.[3,4]

Risk factors predisposing to aminoglycoside nephrotoxicity:

1. Unalterable factors: age, preexisting renal disease or dysfunction
2. Potentially alterable factors: concomitant use of diuretics, or other nephrotoxic agents, radio contrast exposure, effective circulating volume depletion.

## Aminoglycoside Usage in Renal Failure

Aminoglycosides are hydrophilic polar compounds that distribute mainly into the extra cellular fluid. A lower volume of distribution has been described in patients with renal failure as with other drugs with low protein binding such as digoxin. This may be due to the displacement of the drug from tissue binding sites by retained products such as urea. As mentioned earlier, aminoglycosides antibiotics exhibit rapid concentration dependent bacterial killing and have significant postantibiotic effect, thereby facilitating single daily dosing. Meta analyses comparing multiple vs single daily dosing have shown similar antibacterial efficacy and the potential for lower nephrotoxicity with single daily dosing which is currently advocated.[5–7] Because aminoglycosides have a narrow therapeutic index, optimization of therapy to minimize risk of toxicity to residual renal function or vestibular toxicity is important for patients with renal failure who have prolonged exposure to the drug.

Tables 1 and 2 show the daily doses of aminoglycosides in adults with dosing adjusted for creatinine clearance. In patients with normal renal functions, defined peak levels of 5-10 mg/L and trough levels <2 mg/L are considered therapeutic for gentamicin, netilmicin and tobramycin. The target peak and trough therapeutic levels for amikacin are 15-20 mg/L and <5 mg/L, respectively.[10] The well known nephrotoxic potential of aminoglycosides has lead physicians to reduce the dosage of the drug. The risk of insufficient bactericidal effect as a result of under dosing has been demonstrated in renal failure patients.[11] Target peak levels of 5-10 mg/L as desired in normal subjects but slightly higher trough levels of 2.5-5 mg/L to achieve optimum efficacy has been advised.

**Table 1:** Single daily dosing of aminoglycosides with dosing intervals based on creatinine clearance[8,9]

Drug	Dosage (mg/kg)	Ccrl > 60 (ml/min)	Ccrl 40-59 (ml/min)	Ccrl 20-39 (ml/min)
Amikacin	15	Every 24 hrs	36 hrs	48 hrs
Gentamicin	5-7	Every 24 hrs	36 hrs	48 hrs
Netilmicin	5-7	Every 24 hrs	36 hrs	48 hrs
Tobramycin	5-7	Every 24 hrs	36 hrs	48 hrs

**Table 2:** Multiple dosing in patients with GFR < 20 ml/min

Drug	Loading dose (mg/kg)	Maintenance dose (mg/kg)	Dosing time interval (Crcl < 20 ml/min)
Amikacin	7.5	7.5	Every 48 hrs
Gentamicin	2-3	1.7	Every 48 hrs
Netilmicin	2-3	1.7	Every 48 hrs
Tobramycin	2-3	1.7	Every 48 hrs

## Pharmacokinetics of Aminoglycosides on Hemodialysis

A large variability in aminoglycoside pharmacokinetic parameters exists in patients on intermittent hemodialysis. Several studies[12–17] have evaluated gentamicin pharmacokinetics in hemodialysis patients. The serum half-life decreases by 10-fold on dialysis compared to the interdialytic period.[12] In the study by Dager *et al*., 167 patients receiving 216 courses of aminoglycosides were evaluated.[14] The mean extrapolated peak concentrations and trough levels were 7.7 ± 1.6 and 3.9 ± 1.2 mg/L, respectively. The authors recommended that targeting peak concentrations of 7-10 mg/L and trough levels of 3.5-5 mg/L was successful in eradicating infections. The removal of gentamicin by different membranes has been evaluated. The removal of gentamicin by dialysers is highly variable and differs according to membrane permeability, length of dialysis sessions, hemodialysis operating characteristics, patient characteristics, delivered Kt/V, etc. Amin *et al*.,[18] determined the pharmacokinetics of gentamicin using polysulphone Fresenius F 80 membrane in eight patients undergoing chronic hemodialysis. The amount of gentamicin recovered was 64.3 ± 14.4 mg with a clearance of 116 ± 9 ml/min while the intradialytic t^½^ was 2.24 ± 0.83 hrs. A similar volume of gentamicin removal has also been described with cellulose acetate membrane[19] with dialytic clearance of 103.5 ml/min (range 87.2-132.7 ml/min).

Gentamicin is a middle molecule (MW 500-2000 KD) and the clearance is higher with high efficiency dialysers. The clearance of gentamicin, however, does not correlate with urea reduction ratios or Kt/V which is conventionally used as measure of dialytic adequacy.[17] Creatinine dialytic clearance was a better indicator of gentamicin dialytic clearance and further studies investigating the relationship between measures of dialytic adequacy and gentamicin removal during dialysis for determination of dosing are necessary.

A substantial rebound of gentamicin concentrations is noted after discontinuation of hemodialysis. The degree of rebound is variable ranging from 0-70% with a mean of 38.7%[17] and occurred approximately 1.5-3 hrs after cessation of hemodialysis. Hence, supplemental doses of gentamicin based on immediate postdialysis concentrations could be overestimated if postdialysis rebound concentrations are not taken into consideration.

Current dosing guidelines for gentamicin use in patients on hemodialysis suggest that one half of the ‘total dose’ should be given after dialysis.[16] The concept of postdialysis dosing of gentamicin has been challenged with traditional dosing after dialysis increasing trough levels of the drug thereby increasing toxicity. The traditional gentamicin regimens used in patients with normal renal functions aim at achieving peak concentrations ≥8 ml/L and trough levels ≤2 mg/L. No data exists in dialysis patients defining appropriate peak and trough levels. It has been proposed[17] that predialysis dosing with higher doses of aminoglycosides is likely to be more effective at achieving target peak concentrations and is possibly less toxic than postdialysis dosing. Predialysis dosing also has a higher probability of achieving target C_max_ with acceptable AUC than postdialysis dosing.

## Pharmacokinetics in Patients on Continuous Ambulatory Peritoneal Dialysis

Gram negative organisms account for approximately 25% of culture positive peritonitis episodes and amino glycosides are an important therapeutic modality in the treatment of peritonitis. The ISPD guidelines for the treatment of peritonitis recommends IP dosing to IV dosing in continuous ambulant peritoneal dialysis (CAPD) since IP dosing results in very high local levels of antibiotics.[20] Table 3 summarizes the ISPD guidelines for the intraperitoneal use of aminoglycosides in the treatment of gram negative peritonitis.[21] Once daily (40 mg gentamicin in 2L) has been found to be as effective as dosing in each exchange (10 mg/2L, 4 exchanges/ day). Intravenous administration of amikacin in a single dose of 7.5 mg/ kg results in therapeutic concentration of >4 mcg/ml in serum and dialysate for up to 72 hours.[22] However, the variability in bioavailability during peritonitis makes the use of intravenous route less certain for single dose therapy. There is no convincing evidence that short courses of aminoglycosides harm residual renal function.[23]

**Table 3:** Intraperitoneal aminoglycoside dosing recommendations for continuous ambulatory peritoneal dialysis patients. Dosing increased by 25% in patients with residual renal function (defined as urine output >100 ml/day)

Drug	Intermittent (once daily)	Continuous (mg/L, all exchanges)
Amikacin	2 mg/kg	LD 25, MD12
Gentamicin	0.6 mg/kg	LD 8, MD 4
Netilmicin	0.6 mg/kg	LD 8, MD 4
Tobramycin	0.6 mg/kg	LD 8, MD 4

LD = Loading dose; MD = Maintenance dose

## Pharmacokinetics in Patients on Continuous Renal Replacement Therapy

Continuous renal replacement therapy (CRRT), particularly continuous veno-venous hemofiltration (CVVH), and continuous veno-venous hemodiafiltration (CVVHDF) are gaining increasing relevance in the routine clinical management of intensive care unit (ICU) patients with acute renal failure (ARF). Extracorporeal clearance significantly alters the pharmacokinetic behavior of drugs and this may lead to the risk of under dosing of antibiotics leading to therapeutic failure and breakthrough resistance in critically ill patients. Removal of solutes during RRT occurs by two different physiochemical processes namely diffusion and convection. While intermittent hemodialysis (IHD) is essentially a diffusive technique, CVVH is a convective one and CVVHDF is a combination of both. The efficiency of drug removal is expected to be CVVHDF > CVVH > IHD.

However, CRRT clearance depends on the operating condition and the physicochemical properties of the drug. Hydrophilic antibiotics, such as aminoglycosides, are cleared in a consistent manner and the extent of the drug removal is directly proportional to the dialyzer surface area and the mode of replacement administration (pre or postdilution), ultra filtration and dialysate flow rates.

The pharmacokinetic profile of aminoglycosides during CVVH has been studied. In CVVH with a filtration rate of 10 ml/min, the clearance of amikacin is similar to that in a patient not receiving CVVH. However, ultrafiltration rates of >1000 ml/hr contribute to a significant total body clearance. Extracorporeal clearance shortened the mean plasma half life to eight hours and augmented total body clearance by 2-3 fold.[24,25] Hence, frequent plasma level monitoring is therefore necessary in patients undergoing CVVH.

## Conclusion

In view of reduced renal excretion, aminoglycosides require dosage modification in patients with renal failure. The traditional concept of conventional postdialysis dosing in patients on hemodialysis needs to be revised in favor of higher predialysis doses to maintain effective bactericidal activity. Regular monitoring of serum level and adjustment of doses accordingly is necessary, and this is particularly relevant in patients undergoing continuous renal replacement therapy to maintain optimum bactericidal efficacy.
